# Quality of life questionnaire predicts poor exercise capacity only in HFpEF and not in HFrEF

**DOI:** 10.1186/s12872-017-0705-0

**Published:** 2017-10-17

**Authors:** Artan Ahmeti, Michael Y. Henein, Pranvera Ibrahimi, Shpend Elezi, Edmond Haliti, Afrim Poniku, Arlind Batalli, Gani Bajraktari

**Affiliations:** 1 Clinic of Cardiology, University Clinical Centre of Kosova, Rrethi i Spitalit, P.N, 10000 Prishtina, Kosovo; 2 Medical Faculty, University of Prishtina, Prishtina, Kosovo; 3 Department of Public Health and Clinical Medicine, Umeå University and Heart Centre, Umeå, Sweden; 4 Molecular & Clinical Sciences Research Institute, St George University London, London, UK

**Keywords:** Heart failure, The Minnesota Living with Heart Failure Questionnaire, Exercise capacity, 6 min walk test, Echocardiography, Quality of life

## Abstract

**Background:**

The Minnesota Living with Heart Failure Questionnaire (MLHFQ) is the most widely used measure of quality of life (QoL) in HF patients. This prospective study aimed to assess the relationship between QoL and exercise capacity in HF patients.

**Methods:**

The study subjects were 118 consecutive patients with chronic HF (62 ± 10 years, 57 females, in NYHA I-III). Patients answered a MLHFQ questionnaire in the same day of complete clinical, biochemical and echocardiographic assessment. They also underwent a 5 min walk test (6-MWT), in the same day, which grouped them into; Group I: ≤ 300 m and Group II: >300 m. In addition, left ventricular (LV) ejection fraction (EF), divided them into: Group A, with preserved EF (HFpEF) and Group B with reduced EF (HFrEF).

**Results:**

The mean MLHFQ total scale score was 48 (±17). The total scale, and the physical and emotional functional MLHFQ scores did not differ between HFpEF and HFpEF. Group I patients were older (*p* **=** 0.003), had higher NYHA functional class (*p* **=** 0.002), faster baseline heart rate (*p* = 0.006), higher prevalence of smoking (*p* = 0.015), higher global, physical and emotional MLHFQ scores (*p* < 0.001, for all), larger left atrial (LA) diameter (*p* **=** 0.001), shorter LV filling time (*p* = 0.027), higher E/e’ ratio (0.02), shorter isovolumic relaxation time (*p* = 0.028), lower septal a’ (*p* = 0.019) and s’ (*p* = 0.023), compared to Group II.

Independent predictors of 6-MWT distance for the group as a whole were increased MLHFQ total score (*p* = 0.005), older age (*p* = 0.035), and diabetes (*p* = 0.045), in HFpEF were total MLHFQ (*p* = 0.007) and diabetes (p = 0.045) but in HFrEF were only LA enlargement (p = 0.005) and age (*p* = 0.013. A total MLHFQ score of 48.5 had a sensitivity of 67% and specificity of 63% (AUC on ROC analysis of 72%) for limited exercise performance in HF patients.

**Conclusions:**

Quality of life, assessment by MLHFQ, is the best correlate of exercise capacity measured by 6-MWT, particularly in HFpEF patients. Despite worse ejection fraction in HFrEF, signs of raised LA pressure independently determine exercise capacity in these patients.

**Electronic supplementary material:**

The online version of this article (10.1186/s12872-017-0705-0) contains supplementary material, which is available to authorized users.

## Background

Heart failure (HF) represents end stage heart disease irrespective of the underlying etiology, and is acknowledged as a major cause of mortality and morbidity [1, 2]. Exercise intolerance and impaired quality of life (QoL) reflect poor prognosis in HF patients, and are considered the hallmark of disease severity, irrespective of left ventricular (LV) ejection fraction (EF) [3–7]. Treatment of HF aims at improving the clinical status, the functional capacity and QoL, as well as reducing mortality and hospitalizations [8]. Recently, QoL improvement has been shown as one of the most important treatment goals in HF, particularly with the documented increase in life expectancy [9, 10]. Moreover, bearing in mind the expected short life expectancy in these patients, QoL seems to be an important objective that needs to always be addressed [11–13].

The QoL in HF is commonly assessed by the Minnesota Living with Heart Failure questionnaire (MLHFQ) [14] and the Quality of Life with Heart Failure questionnaire (QLHF) [15, 16]. We have translated these two questionnaires into Albanian language and used them in Kosovo Heart Failure Patients [17]. The relationship between QoL and other demographic parameters proved controversial with some studies showing that older age is associated with lower QoL and others failing to show similar relationships [18–21]. Also, the relationship between QoL and other parameters, such as gender and race, remain controversial [22, 23]. Few studies investigated the relationship of QoL with exercise capacity and breathlessness, which showed that QoL correlated with limited exercise and higher NYHA class [24, 25]. But, in those studies the relationship of QoL and LV EF remains not certain [25–27]. Therefore, the aim of this prospective study was to assess the relationship between MLHFQ and exercise capacity in HF patients.

## Methods

### Study population

We studied 118 consecutive patients with a clinical diagnosis of congestive HF (age 62 ± 10 years, 57 female) with ischemic or non-ischemic aetiology, who were in New York Heart Association (NYHA) functional class I-III, and were referred to the Clinic of Cardiology, University Clinical Centre of Kosovo, between December 2014 and September 2016. At the time of the study all patients were on full cardiac medications, optimized at least 2 weeks prior to enrollment. Patients with NYHA class IV, those with limited physical activity due to factors other than cardiac symptoms (e.g. arthritis), with more than mild renal or hepatic failure, with chronic obstructive pulmonary disease, with recent acute coronary syndrome, stroke, psychological or psychiatric disorders, or those with severe anemia, were excluded from the study. All patients signed a written informed consent to participate in the study, which was approved by the Ethics Committee of the Medical Faculty, University of Prishtina. This study was supported and monitored by Kosovo Society of Cardiology [27], which is trying to implement European Society of Cardiology guidelines and other current diagnostic and therapeutic recommendations.

### Data collection

A detailed history and clinical assessment were obtained in all patients. Routine biochemical tests, including hemoglobin, lipid profile, blood glucose level and kidney function, were also performed in all study patients. Estimated body mass index (BMI) was calculated from weight and height measurements. Waist and hip measurements were also made and waist/hip ratio was calculated.

### Quality of life assessment

The MLHFQ contains 21 questions, whose aim is to determine how HF affects the physical, psychological and socioeconomic conditions of the patients (Additional file 1 Table S1). The questions refer to the signs and symptoms of HF, social relationships, physical and sexual activity, work and emotions [14] and assesses how HF affected the patient’s life during the previous month. The MLHFQ has a scoring range of 0 for no impairment to 105 for maximum impairment. The questions cover symptoms and signs relevant to HF, physical activity, social interaction, sexual activity, work, and emotions. Three scores were determined: an overall score (21 items, 0–105), the physical dimension (8 items, 0–40), and the emotional dimension (5 items, 0–25), with the highest scores reflecting the worse QoL. The scale of answers to each question ranges from 0 (none) to 5 (very much), where 0 represented no limitation and 105 represented maximal limitation.

### Echocardiographic examination

A single operator performed all echocardiographic examinations using a Philips Intelligent E-33 system with a multi-frequency transducer, and harmonic imaging as appropriate. Using conventional landmarks and recommendations of the American Society of Echocardiography and European Association of Echocardiography [28, 29] we obtained all measurements including, interventricular septal (IVS) thickness, posterior wall (PW) thickness, and LV dimensions, LV volumes and EF using the modified Simpson’s method and left ventricular mass (LVM) using Devereux formula [30].

Ventricular long axis motion was also studied using conventional methods previously described [31], from which the following measurements were obtained; total amplitude as the mitral annular plane systolic excursion (MAPSE) and the tricuspid plane systolic excursion (TAPSE), and long axis myocardial velocities in systole (s’), early (e’) and late (a’) diastole. Mean value of the lateral and septal e’ velocities was also calculated. LV diastolic function was assessed from spectral Doppler recordings, from which LV early (E wave), late (A wave) diastolic velocities, E/A ratio and E/e’ (mean lateral and septal) ratio were all calculated. Finally, LV isovolumic relaxation time (IVRT) was measured. LV filling pattern was considered ‘restrictive’ when E/A ratio was >2.0, E wave deceleration time < 140 ms and the LA trasverse diameter was >40 mm [33]. LA diameter and volumes were measured, according to the guidelines of the American Society of Echocardiography and European Association of Echocardiography [29], maximal volume (LAV max) at the end systole and LA minimal volume (LAV min) at end diastole. LA total emptying fraction was calculated using the formula [32]:

$$ \mathrm{LA}\ \mathrm{total}\  \mathrm{emptying}\  \mathrm{fraction}=\mathrm{LAV}\ \max \hbox{--} \mathrm{LA}\mathrm{V}\ \min /\mathrm{LAV}\ \max\ \mathrm{x}\ 100 $$

### Measurements of LV dyssynchrony

Indirect assessment of LV dyssynchrony was obtained by measuring total isovolumic time (t-IVT), Tei Index and LV-RV pre-ejection time delay, a spreviously described [33] using total LV filling time and ejection times. Total isovolumic time (t-IVT) was calculated as 60 - (total ejection time + total filling time) and was expressed in s/min [34]. Tei index was calculated as the ratio between t-IVT and ejection time [35].

Mitral and tricuspid regurgitation severity were assessed by colour and continuous wave Doppler and was graded as mild, moderate, or severe according to the relative jet area to that of the left atrium (LA) in line with the recommendations of the American Society of Echocardiography [36]. Retrograde trans-tricuspid pressure drop >35 mmHg was taken as an evidence for pulmonary hypertension [28]. All M-mode and Doppler recordings were made at a fast speed of 100 mm/s with a superimposed ECG (lead II). From the pulmonary artery flow recordings pulmonary artery acceleration time (PAAT) [37]. The LV outflow tract (LVOT) diameter and area were measured [38] in order to calculate the average velocity time integral (VTI) and the stroke volume (SV) [39].

### NT-pro BNP measurement

Blood was taken from an antecubital vein in the morning, sober and after staying extended for 20 min. Blood samples were collected into tubes containing potassium ethylenediaminetetraacetic acid (EDTA) (1 g/L plasma) and N-terminal proBNP were calculated with the Cobas Elecsys E411 analyzer (range 5–35,000 pg/mL) using chemiluminescent immunoassay kit (Roche Diagnostics, Grenach -Wyhlen, Germany).

### Six minute walk test

Within 24 h of the echocardiographic examination a 6-MWT was performed on a level hallway surface and was administered by a specialized nurse blinded to the results of the echocardiogram. According to the method of Gyatt et al. [40] patients were informed of the purpose and protocol of the 6-MWT, which was conducted in a standardized fashion without interrupting patient’s regular medications [41]. A 15 m flat, obstacle-free corridor was used and patients were instructed to walk as far as they can, turning 180° after they had reached the end of the corridor, during the allocated time of 6 min. Patients walked unaccompanied so as not to influence walking speed. At the end of the 6 min the supervising nurse measured the total distance walked by the patient.

### Statistical analysis

Data are presented as mean ± SD or proportions (% of patients). Continuous data was compared with two-tailed unpaired Student’s *t* test and discrete data with Chi-square test. Correlations were tested with Pearson coefficients. Predictors of 6-MWT distance were identified with univariate analysis and multivariate logistic regression was performed using the step-wise method, a significant difference was defined as *P* < 0.05 (2-tailed). Patients were divided according to their ability to walk >300 m into good and limited exercise performance groups [42], and were compared using unpaired Student *t*-test.

## Results

The baseline characteristics of the study population are presented in Table 1. All 118 patients completed the MLHFQ. Patients mean age was 62 ± 9.8 years, and 48% were women. The most common comorbidities were hypertension (67%) and diabetes mellitus (27%) and 30% were smokers. Mean 6-MWT distance was 315 ± 115 m, and 47% were in NYHA class II. Table 2 presents baseline echocardiographic variables.

**Table 1:** Baseline characteristics of the study patients

Variable	Means ± SD
Age (years)	62 ± 9.8
BMI (kg/m^2^)	29 ± 3.8
Waist/hip ratio	0.96 ± 0.1
HR (beat/min)	83 ± 19
Diabetes mellitus (%)	27
Arterial hypertension (%)	67
Smoking (%)	30
LBBB (%)	14
NYHA class I, II, III (%)	30, 47, 23
Sinus rhythm (%)	80
B-blockers (%)	78
ACEi (%)	81
Diuretic (%)	76
Ca-blockers (%)	12
Aspirin (%)	77
Oral anticoagulants (%)	22
6MWT(m)	315 ± 115
Hemoglobin (g/dL)	12.6 ± 1.7
Creatinine (μmol/l)	96 ± 45
NT-ProBNP (pg/mL)	3630 ± 3742
MLHFQ - total score	48 ± 17
MLHFQ - physical score	24 ± 9
MLHFQ - emotional score	9 ± 5

*BMI* body mass index, *BUN* blood urea nitrogen, *SBP* systolic blood pressure, *DBP* diastolic blood pressure, *HR* heart rate, *NYHA* New York heart association, *ACEi* angiotensin converting enzyme inhibitors, *Ca-blockers* Calcium channel blockers, *NT-ProBNP* N-terminal pro b-type natriuretic peptide, *LBBB* left bundle branch block, *WBC* white blood cell, *6MWT* 6 min walking test, *MLHFQ* minnesota living with heart failure questionnaire

**Table 2:** Comparison of quality of life between patients HFpEF and HFrEF

Variable	HFpEF	HFrEF	*P* value
	(*n* = 59)	(*n* = 59)	
MLHFQ total	47 ± 18	50 ± 16	0.328
MFHFQ physical	23 ± 9	26 ± 8	0.066
MFHFQ emotional	9 ± 5	9 ± 4	0.521

*MLHFQ* minnesota living with heart failure questioners

The score of total MLHFQ scale was 48 ± 17, whereas the physical and emotional MLHFQ subscales scores were 24 ± 9 and 9 ± 5, respectively (Table 1). The total physical and emotional MLHFQ subscale scores were not different in patients with HF and preserved EF (HFpEF) compared to those with HF and reduced EF (HFrEF) (Table 2).

Five of 59 (10%) patients with HFpEF had AF, compared to 13 of 59 (22%) patients with HFrEF (*p* = 0.07). LA diameter was significantly larger in AF patients compared to non-AF patients in HFrEF patients (*p* = 0.001), but not in HFpEF (*p* = 0.123). However, the 6-MWT distance was not significant in both subgroups.

### Patients with limited exercise vs. preserved exercise capacity (Tables 3 and 4)

Patients with limited exercise, who walked <300 m during 6-MWT, were older (*p* **=** 0.003), had higher NYHA functional class (*p* **=** 0.002), faster baseline heart rate (*p* = 0.006), higher prevalence of smoking (*p* = 0.015), and higher global, physical and emotional MLHFQ scores (*p* < 0.001, for all), compared to those with good exercise capacity. Patients with limited exercise also had larger LA diameter (*p* **=** 0.001), shorter LV FT (*p* = 0.027), smaller septal MAPSE (*p* = 0.037), higher E/e’ ratio (0.020), shorter IVRT (*p* = 0.028), PAAT (*p* = 0.005), lower septal a’ (*p* = 0.019) and s’ (*p* = 0.023), compared to those with preserved exercise capacity. All other clinical and echocardiographic parameters were not significantly different between two groups.

**Table 3:** Comparison of clinical and biochemical data between patients with limited exercise vs. preserved exercise capacity (6-min walk distance)

Variable	6MWT > 300 m	6MWT <300 m	*P* value
	(*n* = 76)	(*n* = 42)	
Age (years)	60 ± 9.5	66 ± 9	0.003
Smoking (%)	28	33	0.516
Diabetes (%)	20	41	0.015
Arterial hypertension (%)	66	69	0.839
LBBB (%)	10	21	0.169
Waist/hips ratio	0.95 ± 0.1	0.98 ± 0.1	0.036
BMI (kg/m^2^)	29 ± 4.1	28 ± 3.8	0.730
BSA (m^2^)	1.2 ± 0.2	1.1 ± 0.2	0.046
NYHA class			0.002^a^
NYHA class I, II, III (%)	38, 47, 15	14, 45, 41	
HFpEF (%)	46	57	0.249
Fasting glucose (mmol/L)	6.4 ± 2.5	7.8 ± 2.9	0.013
Creatinine (μmol/L)	97.6 ± 54	93.0 ± 21	0.604
Hemoglobin (g/dL)	12.7 ± 1.6	12.3 ± 2.0	0.203
HR (beats/min)	72 ± 15	80 ± 13	0.006
NT-ProBNP (pg/mL	1510 ± 4146	1832 ± 2907	0.66
MLHFQ total	43.8 ± 16.9	56.9 ± 14.4	<0.001
MLHFQ physical	22.1 ± 9	27.6 ± 6.9	0.001
MLHFQ emotional	8.0 ± 4.3	10.9 ± 4.3	0.001

*BMI* body mass index, *BSA* body surface area, *HR* heart rate, *NYHA* New York heart association, *LBBB* left bundle branch block, *NT-ProBNP N*-terminal pro b-type natriuretic peptide, *HFpEF* heart failure with a preserved ejection fraction, *MLHFQ* minnesota living with heart failure questionnaire

^a^NYHA class significance between study groups

**Table 4:** Comparison of echocardiographic data between patients with limited exercise vs. preserved exercise capacity (6-min walk distance)

Variable	6MWT > 300 m	6MWT <300 m	*P* value
	(*n* = 76)	(*n* = 42)	
LV EF (%)	48 ± 15	45 ± 15	0.445
IVS (cm)	1.12 ± 0.2	1.11 ± 0.1	0.986
LA diameter (cm)	4.2 ± 0.7	4.7 ± 0.9	0.001
LV EDD (cm)	5.7 ± 0.8	5.9 ± 1.1	0.271
LV ESD (cm)	4.2 ± 1.1	4.5 ± 1.3	0.261
Lateral MAPSE (cm)	1.3 ± 0.9	1.2 ± 1.1	0.786
Septal MAPSE (cm)	1.0 ± 0.3	0.9 ± 0.3	0.037
TAPSE (cm)	2.2 ± 2.3	2.1 ± 2.6	0.670
LV posterior wall (cm)	1.1 ± 0.4	1.0 ± 0.1	0.913
E/A ratio	1.0 ± 0.6	1.1 ± 0.8	0.325
FT (ms)	431 ± 138	379 ± 105	0.027
IVRT (ms)	132 ± 42	111 ± 34	0.028
PAAT (ms)	114 ± 23	100 ± 22	0.005
E/e’ ratio	10 ± 4.1	13 ± 8.0	0.020
Lareral e’ (cm/s)	6.1 ± 2.5	6.0 ± 2.7	0.881
Lateral a’ (cm/s)	8.2 ± 3.7	7.6 ± 3.5	0.393
Lateral s’ (cm/s)	5.5 ± 1.5	4.9 ± 1.6	0.074
Septal e’ (cm/s)	5.1 ± 2.2	4.6 ± 2.1	0.295
Septal a’ (cm/s)	7.5 ± 2.4	6.4 ± 1.9	0.019
Septal s’ (cm/s)	4.6 ± 1.6	4.0 ± 1.1	0.023
LA EF (%)	49 ± 17	45 ± 16	0.313

*LV* left ventricle*, EDD* end-diastolic dimension*, ESD* end-systolic dimension*, FT* filling time*, ET* Ejection time*, IVS* interventricular septum*, IVRT* isovolemic relaxation time*,* e’ early diastolic myocardial velocity*, s*’ systolic myocardial velocity*, LA* left atrium*, LA EF* Left atrial emptying fraction*, A* atrial diastolic velocity*, E* early diastolic filling velocity*, PAAT* pulmonary artery acceleration time*, MAPSE* mitral annular plane systolic excursion*, TAPSE* tricuspid annular plane systolic excursion

### Relationship of total MLHFQ with clinical, biochemical and echocardiographic variables (Table 5)

**Fig. 1:**
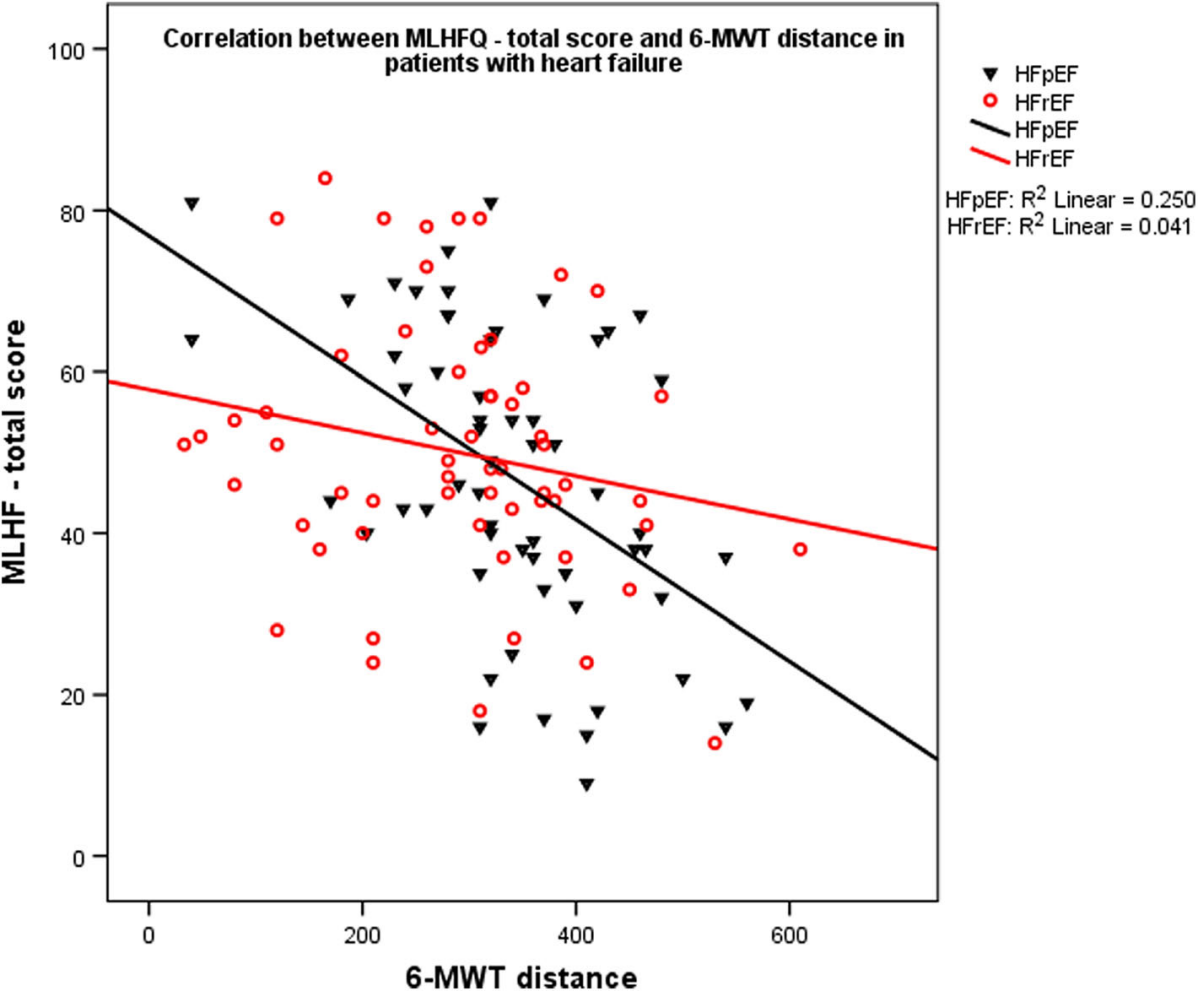
Correlation between total MLHFQ score and 6-MWT distance in patients with heart failure

In the patients’ group as a whole, total MLHFQ score had strong correlation with 6-MWT distance, lateral s’ (*p* < 0.001 for both), good correlation with LVMI (*p* = 0.001) and with lateral MAPSE (*p* = 0.009), and weak correlation with hemoglobin level (*p* = 0.024). In HFpEF, total MLHFQ score had strong correlation with 6-MWT distance (p < 0.001, Fig. 1), and weak correlation with lateral s’ (*p* = 0.014), LVMI (*p* = 0.027) and with hemoglobin level (*p* = 0.016), whereas in HFrEF patients it has only a weak correlation with lateral s’ (*p* = 0.03), LVMI (*p* = 0.027), lateral MAPSE (*p* = 0.027) and with E/A ratio (*p* = 0.047).

**Table 5:** Correlation of MLHFQ total score in HF patients with clinical, biochemical and echocardiographic variables in study patients

Variable	All study patients (*n* = 118)		HFpEF (*n* = 59)		HFrEF (*n* = 59)	
	R	p	r	P	R	P
6-MWT	−0.359	<0.001	−0.500	<0.000	−0.203	0.123
Age	0.081	0.281	0.013	0.922	0.144	0.278
Creatinine	−0.076	0.418	0.093	0.481	−0.178	0.181
Hemoglobin	−0.208	0.024	−0.312	0.016	−0.136	0.304
BMI	0.119	0.198	0.179	0.175	0.073	0.582
BSA	−0.139	0.134	−0.171	0.194	−0.104	0.433
LVMI	0.292	0.001	0.289	0.027	0.287	0.027
LA diameter	0.112	0.226	0.108	0.415	0.070	0.598
LV EF	−0.081	0.384	0.117	0.378	−0.179	0.174
E/A	0.165	0.080	−0.092	0.489	0.269	0.047
Lateral MAPSE	−0.245	0.009	−0.160	0.229	−0.308	0.021
Lateral s’	−0.306	<0.001	−0.319	0.014	−0.282	0.030
E/e’	0.173	0.092	0.026	0.855	0.263	0.089
Septal s’	−0.137	0.179	−0.111	0.426	−0.094	0.544

*MLHFQ* minnesota living with heart failure questionnaire*, 6-MWT* 6 min walking test*, BMI* body mass index*, BSA* body surface area*, EDD* end-diastolic dimension*, MAPSE* mitral annular plane systolic excursion*, A* atrial diastolic velocity*, E* early diastolic filling*,* velocity*, e*’ early diastolic myocardial velocity*, s*’ systolic myocardial velocity*, LVMI* left ventricular mass index

### Predictors of limited 6-MWT distance in HF patients (Table 6)

#### Predictors of limited 6 MWT distance in all HF patients

**Fig. 2:**
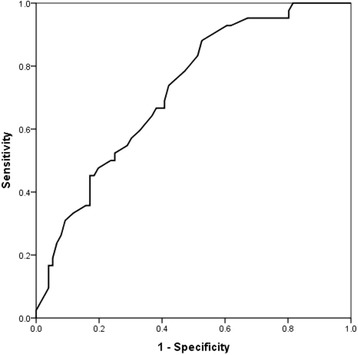
ROC-curve of MLHFQ - total score in predicting poor exercise performance on 6-min walk test in patients with heart failure

In univariate analysis, total MLHFQ (*p* < 0.001), physical MLHFQ (*p* = 0.002), emotional MLHFQ (*p* = 0.002), age (*p* = 0.005), diabetes (*p* = 0.017), atrial fibrillation (*p* = 0.006), LA diameter (*p* = 0.001), IVRT (*p* = 0.047), PAAT (*p* = 0.008), septal MAPSE (p = 0.04), E/e’ (*p* = 0.029), septal a’ (*p* = 0.033), and septal s’ (*p* = 0.041), predicted limited 6 MWT distance. In multivariate analysis, only total MLHFQ score (*p* = 0.005), age (*p* = 0.035) and the diabetes (*p* = 0.045) remained independent predictors of limited 6-MWT distance. A total MLHFQ score of 48.5 had a sensitivity of 67% and specificity of 63% (AUC on ROC analysis of 72%) for predicting limited exercise performance (Fig. 2).

**Table 6:** Predictors of limited exercise in HF patients

Variable	*Univariate predictors*			*Multivariate predictors*		
	OR	CI 95%	*P* value	OR	CI 95%	*P* value
Whole HF study patients						
MLHFQ - total score	1.053	(1.025–1.081)	<0.001	1.080	(1.023–1.140)	0.005
MLHFQ - physical score	1.085	(1.031–1.141)	0.002			
MLHFQ - emotional score	1.163	(1.060–1.277)	0.001			
Age	1.069	(1.021–1.120)	0.005	1.101	(1.007–1.203)	0.035
Gender	0.576	(0.269–1.232)	0.155			
Diabetes mellitus	2.765	(1.199–6.379)	0.017	4.876	(1.037–22.94)	0.045
LA diameter	2.500	(1.467–4.260)	0.001			
FT	0.996	(0.993–1.000)	0.046			
IVRT	0.984	(0.969–1.000)	0.047			
PAAT	0.972	(0.952–0.993)	0.008			
Septal MAPSE	0.235	(0.059–0.939)	0.040			
E/e’	1.090	(1.009–1.177)	0.029			
Septal a’	0.787	(0.632–0.981)	0.033			
Septal s’	0.663	(0.447–0.983)	0.041			
HFpEF patients						
MLHFQ - total score	1.080	(1.032–1.131)	<0.001	1.137	(1.036–1.249)	0.007
MLHFQ - physical score	1.088	(1.010–1.173)	0.026			
MLHFQ - emotional score	1.213	(1.055–1.396)	0.007			
Diabetes mellitus	3.556	(1.089–11.61)	0.036	26.88	(1.791–400.8)	0.017
Hemoglobin	0.711	(0.514–0.983)	0.039			
NYHA class	3.038	(1.271–7.262)	0.012			
BSA	0.003	(0.000–0.241)	0.009			
LVMI	10.50	(1.007–1.096)	0.023			
Lateral a’	0.768	(0.604–0.978)	0.032			
Lateral s’	0.468	(0.258–0.850)	0.013			
HFrEF patients						
MLHFQ - physical score	1.076	(1.002–1.156)	0.044			
Age	1.071	(1.013–1.132)	0.015	1.113	(1.024–1.209)	0.012
NYHA class	2.501	(1.064–5.881)	0.036			
LVM	1.010	(1.001–1.019)	0.036	1.015	(1.000–1.030)	0.047
LA diameter	3.183	(1.356–7.475)	0.008	7.401	(1.821–30.08)	0.005
LV EDD	1.070	(1.002–1.143)	0.044			

*MLHFQ* Minnesota living with heart failure questionnaire*, NYHA* New York Heart Association*, BSA* body surface area*, LV* left ventricle*, LVM* left ventricular mass*, LA* left atrium*, EDD* end-diastolic dimension*, FT* filling time*, IVRT* isovolemic relaxation time*, MAPSE* mitral annular plane systolic excursion*, E* early diastolic filling*,* velocity*, e*’ early diastolic myocardial velocity*, s*’ systolic myocardial velocity, *PAAT* pulmonary artery acceleration time, *LVMI* left ventricular mass index, *HFrEF* heart failure with reduced ejection fraction

#### Predictors of limited 6-MWT distance in HFpEF patients

Univariate analysis identified total MLHFQ (*p* = 0.001), physical MLHFQ (*p* = 0.026), emotional MLHFQ (*p* = 0.007), BSA (*p* = 0.009), diabetes (*p* = 0.036), and NYHA class >1 (*p* = 0.012), hemoglobin level (*p* = 0.039), increased LVMI (*p* = 0.023), low lateral s’ (*p* = 0.013) and a’ (*p* = 0.032) as predictors of limited 6-MWT distance. In multivariate analysis, total MLHFQ (*p* = 0.007) and diabetes (*p* = 0.045) independently predicted the limited 6-MWT distance.

#### Predictors of limited 6 MWT distance in HFrEF patients

In univariate analysis, physical MLHFQ (*p* = 0.044), age (*p* = 0.015), NYHA class >1 (*p* = 0.036), LV mass (p = 0.036) and LA diameter (*p* = 0.008), predicted the 6-MWT limited exercise distance. In multivariate analysis, only LA enlargement (*p* = 0.005) and age (*p* = 0.013) remained independent predictors of limited 6-MWT distance.

## Discussion

### Findings

The results of this study analysis can be summarized as follows: 1) the total scale, physical and emotional MLHFQ subscale scores were not different between HFpEF and HFrEF patients. 2) Patients with limited exercise capacity were older, had higher NYHA functional class, faster baseline heart rate, higher prevalence of smoking and higher global, physical and emotional MLHFQ scores, compared to those with good exercise capacity. 3) Patients with limited exercise capacity, also had larger LA, shorter LV FT, worse longitudinal systolic function and raised LV filling pressures, compared to those with preserved exercise capacity. 4) Total MLHFQ score had strong correlation with 6-MWT distance in the patients group as a whole and in HFpEF subgroup, but not in HFrEF. 5) Total MLHFQ score, age and diabetes were the only independent predictors of limited 6-MWT distance in the whole group of patients and in HFpEF subgroup. It was LA enlargement and age which independently predicted limited exercise capacity in HFrEF.

### Results interpretation

MLHFQ irrespective of its components; physical or emotional seems to be a good measure of exercise capacity, since it correlated strongly with the 6-MWT distance in the HF group irrespective of EF. Thus, it could be used to reflect the overall cardiac status, when used to evaluate patients’ response to treatment. It however, does not reflect the underlying cardiac structural or functional disturbances, which contribute to the limited exercise capacity in individual patients, and which might need different treatments. Age seemed to be correlating with limited exercise capacity but nothing can be done about it. On the other hand baseline heart rate proved to be an equally important factor but can be managed by beta blockers [43] or other forms of heart rate controlling medications e.g. Ivabridine [44], or the combination of the two [45]. Furthermore, patients with limited exercise capacity proved to have dilated LA [46, 47], the underlying pathophysiology of which is known to be complicated. It proved to be related to the high filling pressures in some [48] and poor LA emptying, as shown be short LV filling time, in others [49]. In addition to the variety of mechanisms of disturbed physiology, the matter is further complicated by the way patients differ in their response to treatment. While the former group usually responds to LA pressure lowering medications i.e. ACE-inhibitors or A2 blockers [50], the latter respond better to cardiac resynchronization therapy [51]. Finally, it seems that predictors of the limited exercise capacity differed fundamentally according to the cardiac physiology. While specifically the causes of LA enlargement; pressure, mitral regurgitation, stiff LV, etc., that limited patients exercise in HFrEF, the respective reasons were multifactorial including age, diabetes, as well as emotional and physical scores that predicted exercise capacity in HFpEF. The latter finding adheres to what is known about HFpEF in terms of its etiology, comorbidities as well as limited benefit when using conventional guidelines-based treatment recommendations [52]. The lack of an acceptable relationship between LA volume and exercise capacity in HFpEF could be explained by either strict early treatment with vasodilators which reduced cavity pressure and hence volume or less myocardial stiffness compared with HFrEF. Also, despite higher AF prevalence in HFrEF patients compared to HFpEF, our analysis suggest that AF was not necessarily a determinant factor for the difference in relationship between left atrial enlargement and 6-MWT. It seems therefore that more than one factor could contribute to the lack of direct relationship between the LA volume and exercise capacity in HFpEF. It was however not feasible to run a number of permutations and combinations in order to identify the additive value of various individual variables in predicting exercise capacity.

### Clinical implications

Our findings suggest that the MLHFQ correlates with 6MWT distance in heart failure patients as a whole and is able, fairly accurately, to predict those with limited exercise capacity. These findings apply better to patients with HFpEF much more than those with HFrEF in whom clearly signs of raised LA pressures are those which independently determine their limited exercise capacity. These differences support the need for continuing the use of detailed Doppler echocardiographic follow up of heart failure patients in order to better understand the pattern of disturbances that explain symptoms as well as the most accurate treatment option.

### Limitations

Obvious limitations can easily be seen in this study. The small number of patients included in this study limits general application of the findings before results are revalidated in a larger cohort. We consider that further prospective cohort studies with a larger sample size, are undoubtedly needed to strengthen or refute our findings. Speckle tracking ultrasonography to measure the global longitudinal strain, which might be associated with reduced functional capacity in HF patients was not used. However, assessing longitudinal LV function with conventional tools, provided an estimate of other overall longitudinal LV function. We cannot ignore the emotional element in conducting the 6-MWT and patient encouragement to walk faster, although unassisted. We did not assess the reproducibility of the results of the MLHFQ neither the 6-MWT distance, which could have shown significant differences.

## Conclusion

Although the conventionally used MLHFQ, irrespective of its components, correlates closely with the 6-MWT distance in HF patients particularly HFpEF. Raised filling pressures seem to be the strongest independent predictor of limited exercise capacity in HFrEF. These differences might impact treatment options in the two conditions.

## Additional file

Additional file 1: Table S1. The Minnesota Living with Heart Failure Questionnaire. (DOC 29 kb)

## Abbreviations

6-MWT: Minute walk test
A: Atrial diastolic velovity
a’: Atrial myocardial velocity
BMI: Body mass index
BSA: Body surface area
DT: Deceleration time
E: Early diastolic velocity
e’: Early diastolic myocardial velocity
EF: Ejection fraction
FT: Filling time
HF: Heart failure
HFpEF: Heart failure with preserved ejection fraction
HFrEF: Heart failure with reduced ejection fraction
LA EF: Left atrial emptying fraction
LA: Left atrium
LAV max: Left atrial maximal volume
LAV min: Left atrial minimal volume
LV: Left ventricle
LVM: Left ventricular mass
LVMI: Left ventricular mass index
LVPWd: Left ventricular posterior wall in diastole
MAPSE: Mitral annular plane systolic excursion
MLHFQ: The Minnesota Living with Heart Failure Questionnaire
NYHA: New York Heart Association
PA: Pulmonary artery
QoL: Quality of life
RV: Right ventricle
s’: Systolic myocardial velocity
TAPSE: Tricuspidal annular plane systolic excursion
