# Supplementary material for: Quality of life questionnaire predicts poor exercise capacity only in HFpEF and not in HFrEF
Source: BMC Cardiovasc Disord. 2017 Oct 17;17:268. doi: 10.1186/s12872-017-0705-0 (PMC5646144; doi:10.1186/s12872-017-0705-0)
Supplement: Additional file 1: Table S1. — The Minnesota Living with Heart Failure Questionnaire. (DOC 29 kb) [file 12872_2017_705_MOESM1_ESM.doc]

MINNESOTA LIVING WITH HEART FAILURE QUESTIONNAIRE

The following questions ask how much your heart failure (heart condition) affected your life during the past month (4 weeks). After each question, circle the 0, 1, 2, 3, 4 or 5 to show how much your life was affected. If a question does not apply to you, circle the 0 after that question.

**Did your heart failure prevent you from living as you wanted during the past month (4 weeks) by –**

**0 - No Little Much**

1. **very little**

**5- very much**

1. Causing swelling in your ankles or legs? 0 1 2 3 4 5

2. Making you sit or lie down to rest during the day? 0 1 2 3 4 5

3. Making your walking about or climbing stairs difficult? 0 1 2 3 4 5

4. Making your working around the house or yard difficult? 0 1 2 3 4 5

5. Making your going places away from home difficult? 0 1 2 3 4 5

6. Making your sleeping well at night difficult? 0 1 2 3 4 5

7. Making your relating to or doing things with your friends or family difficult? 0 1 2 3 4 5

8. Making your working to earn a living difficult? 0 1 2 3 4 5

9. Making your recreational pastimes, sports or hobbies difficult? 0 1 2 3 4 5

10. Making your sexual activities difficult? 0 1 2 3 4 5

11. Making you eat less of the foods you like? 0 1 2 3 4 5

12. Making you short of breath? 0 1 2 3 4 5

13. Making you tired, fatigued, or low on energy? 0 1 2 3 4 5

14. Making you stay in a hospital? 0 1 2 3 4 5

15. Costing you money for medical care? 0 1 2 3 4 5

16. Giving you side effects from treatments? 0 1 2 3 4 5

17. Making you feel you are a burden to your family or friends? 0 1 2 3 4 5

18. Making you feel a loss of self-control in your life? 0 1 2 3 4 5

19. Making you worry? 0 1 2 3 4 5

20. Making it difficult for you to concentrate or remember things? 0 1 2 3 4 5

21. Making you feel depressed? 0 1 2 3 4 5

_________________________________________________________________________

©1986
